# Season, Body Condition and Developmental Stage Influence the Gut Microbiota of the Sole Living Rhynchocephalian Reptile (*Sphenodon punctatus*)

**DOI:** 10.1002/ece3.71068

**Published:** 2025-04-11

**Authors:** Carmen Hoffbeck, Danielle M. R. L. Middleton, Nicola J. Nelson, Michael W. Taylor

**Affiliations:** ^1^ School of Biological Sciences University of Auckland Auckland New Zealand; ^2^ Manaaki Whenua–Landcare Research Lincoln New Zealand; ^3^ School of Biological Sciences Victoria University of Wellington Wellington New Zealand

**Keywords:** microbiome, reptile, season, tuatara

## Abstract

Seasonality plays a crucial role for many species, especially reptiles. In multiple reptile species, seasonality has been linked to shifts in the gut microbiota, influenced by factors, such as ambient temperature, food availability and shifting host function across different seasons. We tested whether the tuatara, an endemic New Zealand reptile and the sole extant member of the order Rhynchocephalia, maintains a stable gut microbiota over 2 years of sampling across three seasons (summer, autumn, spring) or if the dominant bacterial community varies with season. We found that community diversity changed significantly seasonally, with the most diverse gut community found in the spring. We also found that season significantly influenced beta‐diversity, as did tuatara developmental stage, tuatara body condition and tick abundance. However, there was little evidence for a recurring seasonal bacterial assemblage in 2024 compared with 2023. For tuatara where the same individual was resampled over multiple seasons, bacterial community composition appeared to be most correlated with the time of sampling, with closer temporal samples more similar to one another than samples taken further apart, which was also seen in the significance of the sampling period as a factor explaining variation across all tuatara. We identified bacterial genera that significantly increased or decreased in each season. Despite notable shifts among seasons, particularly in autumn, the tuatara gut microbiota exhibits remarkable persistence over time, including within individuals.

## Graphical abstract

Seasonality plays a crucial role for many species, especially reptiles. We tested whether the tuatara, an endemic New Zealand reptile and the sole extant member of the order Rhynchocephalia, maintains a stable gut microbiota over 2 years of sampling across three seasons (summer, autumn and spring) or if the dominant bacterial community varies with season. We found that community diversity changed significantly seasonally, with the most diverse gut community found in the spring; that season significantly influenced beta‐diversity, as did tuatara life stage, tuatara body condition and tick abundance. However, there was little evidence for a reoccurring seasonal bacterial assemblage in 2024 compared with 2023.

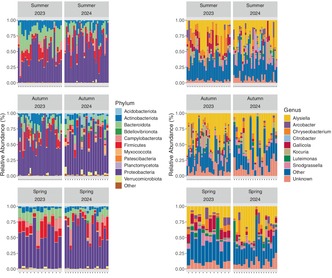

## 1 Introduction

The collection of microorganisms (collectively dubbed the ‘microbiota’) associated with a host animal exhibits complicated community dynamics, with the identity and diversity of species making up the microbiota of the digestive tract influencing how hosts break down food (Cheng et al. 2022; Wilson et al. 2020; Xu and Knight 2015), respond to pathogens (Dillon et al. 2005; Kamada et al. 2013; Pickard et al. 2017), and develop and behave (Ezenwa et al. 2012; Sampson and Mazmanian 2015). Seasonality is a significant ecological factor that alters the gut microbiota in some species. In the wild, seasons dictate both abiotic factors like ambient temperature and humidity and biotic factors, such as food availability, reproductive state and competition. For ectothermic species like reptiles, seasonality is likely even more important, as these animals have less control over their internal temperature (Jessop et al. 2022). Seasons also influence food selection, particularly for omnivorous species. Reptiles might be primarily frugivorous when fruits are readily available, then switch to insects in other seasons (Gao et al. 2023). Some reptiles hibernate or enter a state of torpor, wherein they may eat little to no food for weeks or months at a time (Tang et al. 2019). The influence of these factors on reptiles' gut microbiota at large is poorly understood (Hoffbeck et al. 2023); however, individual species have been shown to be influenced by external temperature (Moeller et al. 2020; Sepulveda and Moeller 2020; Zhang et al. 2022), food selection (Du et al. 2022; Holmes et al. 2019), hibernation (Tang et al. 2019) and seasonal shifts more broadly (Baldo et al. 2023; Gao et al. 2023; Hernández et al. 2024; Jessop et al. 2022).

Long‐term studies sampling the microbiota of wild populations over multiple seasons or years are rare, especially in reptile species. For reptiles where seasonal sampling has been conducted, the gut community largely shows shifts with seasonality, tied to covarying factors like hibernation (Bunker et al. 2022; Tang et al. 2019) and dietary availability (Baldo et al. 2018; Gao et al. 2023; Hernández et al. 2024). The majority of these samples come from lizards, with gut communities dominated by phyla, including Firmicutes and Bacteroidota, across all seasons (Hoffbeck et al. 2023). The strongest shifts in microbes across seasons appear to be linked to fermenting bacteria (Gao et al. 2023; Tang et al. 2019), with implications for how the gut community can adapt to the diet (or lack thereof when hibernating) across different seasons. Across multiple species of lizards, trends in diversity vary. For some species, gut microbial diversity is highest in spring and summer during mating season (Gao et al. 2023), whereas for others, it is lowest during this period of sociality (Bunker et al. 2022). Such differences in seasonal gut diversity persist even among closely related reptile species (Hernández et al. 2024), implying complex links between gut community assembly, diversity and seasonal dynamics.

The tuatara ( *Sphenodon punctatus* ) is an endemic reptile to Aotearoa New Zealand. These unique reptiles are the sole extant representatives of order Rhynchocephalia, having diverged from their closest living relatives (order Squamata) over 200 million years ago (Evans and Jones 2010). Tuatara live only in New Zealand and were extirpated from the mainland following human colonisation (Towns and Daugherty 1994), leaving only 32 populations surviving on small offshore islands (Cree 2014). In addition to their evolutionary status, tuatara are also cold‐adapted reptiles with a low thermal tolerance despite being ectothermic (Besson and Cree 2010) and forage nocturnally in temperatures as low as 7°C (Saint Girons et al. 1980). Tuatara are omnivorous, eating a range of insects, seabirds, plants and other items when available (Fraser 2012). They also experience a period of lessened activity in winter, at which point tuatara decrease foraging and eating until temperatures rise (Mello et al. 2013). Their survival in New Zealand, and therefore the persistence of their order, is threatened by changing temperatures (Mitchell et al. 2008) and invasive predators (Gaze 2001).

The first study of the tuatara gut microbiota revealed that tuatara share relatively few of the bacterial genera common to their closest relatives, the squamates (Hoffbeck et al. 2024a), notably having a low abundance of the genus *Bacteroides*, a critical bacterial genus in the gut of many other reptiles (Hoffbeck et al. 2023, 2025). Tuatara spanning ~1000 km of latitude in sanctuaries across New Zealand share a relatively large core community and are dominated by bacterial genera, such as *Kocuria*, *Chryseobacterium* and *Gallicola* (Hoffbeck et al. 2024a). The high degree of similarity in the bacterial communities of tuatara spanning a large climatic range, and which experience no interaction or migration, may indicate that the gut community of this species is largely stable over time, though all previous sampling was conducted on tuatara from the same genetic population and occurred in the same season. Furthermore, the microbiotas of individual tuatara in captivity can fluctuate, though they remain largely dominated by the same bacterial genera (Hoffbeck et al. 2024b). Individual tuatara body condition and tick abundance were both correlated with significant differences in the gut community (Hoffbeck et al. 2024a), as was the temperature of the site. Along with diversity shifts linked to temperature in wild tuatara, the gut communities of captive tuatara varied among individuals at the beginning of sampling in October but converged to a relatively consistent community by the beginning of summer in January (Hoffbeck et al. 2024b). Whether the tuatara gut community remains stable over seasons and longer time periods, and the extent to which ambient temperature influences the tuatara gut community, remains unknown.

In this study, we sampled a wild population of tuatara over six seasons covering 2 years of summer, autumn and spring. This allowed us to test several hypotheses: (1) the tuatara gut microbiota varies between seasons, (2) the dominant gut microbiota during each season re‐establishes in subsequent samplings and (3) individual variation among tuatara is correlated with individual body condition and tick abundance. This research further informs how the gut microbiota of a unique and important species is influenced by environmental conditions and examines the stochasticity of the tuatara gut microbiota over repeated samplings spanning 2 years.

## 2 Materials and Methods

### 2.1 Sample Collection

Tuatara were sampled at Zealandia Te Māra a Tāne, an ecosanctuary located in Wellington, New Zealand. Zealandia is a fully fenced sanctuary with no invasive predators. Night‐time sampling at this site occurred in January, April and October 2023, and January, April and October 2024, representing the Austral summer, autumn and spring, respectively. Tuatara were not sampled in winter because of their inactivity and consequent difficulty to catch. To obtain a proxy for the gut microbiota, tuatara were captured by hand and sampled via cloacal swabbing. A small cotton‐tipped swab was inserted 20 mm into the cloaca and rotated gently on the cloacal wall before being removed and placed into RNAlater for storage at −20°C. Animal ethics approval was granted by Victoria University of Wellington (Permission #30011), and research approval was given by the Department of Conservation (Authorisation 50,568‐FAU). Each animal was identified by subcutaneous transponder (PIT tag) number or historic toe clipping, and was demarcated by xylene‐free marker pen if no other identifying marks were present to avoid repeat sampling of the same individual during a given season. Some individual tuatara were sampled across multiple seasons, but individuals were not intentionally resampled and individuals without PIT tags or toe clips could not be consistently identified from season to season. Additional information about tuatara sex, life stage, tick count and body condition was obtained as described previously (Hoffbeck et al. 2024a). Briefly, sex and life stage were estimated from secondary sex characteristics. Cutaneous ticks were identified and counted to the nearest five. Tuatara were weighed by spring balance and measured for snout‐vent length (SVL), which was then used to calculate body condition by calculating $\frac{\log\left(\text{weight}\right)}{\log\left(\mathrm{SVL}\right)}$ (Lamar et al. 2022). In total across all seasons, swabs were taken from at least 50 (potentially up to 93) individual tuatara representing 146 samples for analysis. These data included 62 summer samples, 49 autumn samples and 35 spring samples; 120 adults and 26 subadults; and 81 males and 47 females.

### 2.2 DNA Extraction, 16S rRNA Gene Amplification and Sequencing

All swabs were removed from RNAlater, and any DNA present was extracted using a QIAGEN QIAamp Fast DNA Stool Kit, as recommended by the manufacturer for bacterial DNA collection. Though this kit is designed for faecal input, its efficacy for tuatara DNA extraction had been previously determined for a single swab input (Hoffbeck et al. 2024a). For each season, an additional extraction blank was included by extracting a sterile swab. Extracted DNA was stored at −20°C. The near‐entire 16S rRNA gene was amplified using the ONT 27F‐1492R primer pair (Frank et al. 2008) and LongAmp Taq 2X Master Mix. The following thermal cycling conditions were used: initial denaturation at 94°C for 30 s, then 30 cycles of denaturation at 94°C for 30 s, annealing at 55°C for 30 s, and extension at 65°C for 30 s, followed by a final extension at 65°C for 10 min. Amplicon length was checked by 1% agarose gel electrophoresis, and PCR yield was quantified using an EnSpire Multimode Plate Reader. After amplification, 20 μL of PCR product from each sample was purified using Agencourt AMPure XP beads and sequenced on an Oxford Nanopore GridION by Auckland Genomics Ltd. using the 16S Barcoding Kit SQK‐RAB204.

### 2.3 Bioinformatics

Reads selected with Super High Basecalling on Nanopore were retained for analysis. These reads were trimmed using NanoFilt (De Coster et al. 2018) to 1500 bp and quality level 10. Retained reads after trimming were processed using Emu (Curry et al. 2022) with the SILVA‐138 database (Quast et al. 2013) into ‘number of taxonomic units’ or ‘NTUs’, analogous to amplicon sequence variants. Contaminant reads were determined by presence in the extraction blanks, and these were removed prior to analysis using the decontam package in R (Davis et al. 2018) with default threshold 0.1, resulting in the removal of 20 contaminant NTUs. To compare across different sampling depths, each sample was standardised to 1000 sequence reads using the SRS function in the SRS package (Heidrich et al. 2021), resulting in the removal of 11 samples with low sequencing depth (Figure S1). A total of 135 samples across all seasons were retained for downstream analysis.

Bacterial community alpha diversity was calculated using the plot_richness function in phyloseq (McMurdie and Holmes 2013) with the observed richness, Shannon, Simpson and Chao1 diversity metrics, with statistical significance determined by the Wilcoxon test. Beta‐diversity was calculated using the ordinate function in phyloseq with Bray–Curtis dissimilarity and visualised using non‐metric multidimensional scaling (nMDS) and hierarchical clustering using the Ward method. Significance was determined for discrete variables (including sex, developmental stage and season) using the adonis2 PERMANOVA function in vegan (Oksanen et al. 2022) with Bray–Curtis and Jaccard dissimilarity and 9999 permutations. For continuous variables (including tick abundance and body condition), the envfit function in vegan was used to determine significance and visualise effect sizes. Taxonomy was visualised at the phylum and genus levels using ggplot2 (Wickham et al. 2019).

The ‘core’ community was defined as bacteria shared by more than 80% of individuals and was established using the core_members function of the microbiome package (Lahti and Shetty 2019). The core microbiota was assessed both overall and within each season.

## 3 Results

### 3.1 Response of the Gut Microbiota to Differences in Season, Tuatara Developmental Stage and Tuatara Sex

**FIGURE 1:**
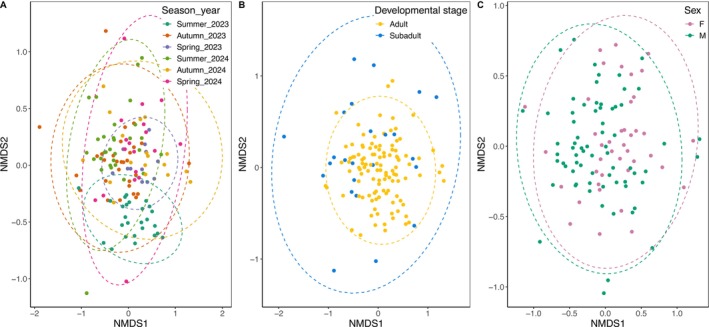
nMDS plot representing Bray–Curtis dissimilarity of the tuatara gut microbiota across (A) seasons, (B) developmental stage and (C) sex.

As hypothesised, season—characterised as either season per se (summer, autumn and spring) or sampling season (summer 2023, autumn 2023, spring 2023, summer 2024, autumn 2024 and spring 2024)—was significantly correlated with different microbiota in the tuatara gut (*R* ^2^_season_ = 5.73, *p*_season_ < 0.0001; *R* ^2^_season and year_ = 9.54, *p*_season and year_ < 0.0001; Table 1). Despite this, all sampling seasons overlapped substantially when visualised using nMDS (Figure 1A). Similarly, both sex and developmental stage of the tuatara explained significant variation in the microbiota (*R* ^2^_stage_ = 3.80, *p*_stage_ < 0.0001; *R* ^2^_sex_ = 3.86, *p*_sex_ < 0.001), though these variables explained less variation than season (Figure 1B,C). Though there were some distinct groupings of these variables, samples did not clearly cluster across tested variables (Figure S5). Overall, most NTUs were shared across variable groups, with more unique NTUs attributed primarily to the group with the larger sample size (Figure S6).

**TABLE 1:** PERMANOVA results based on Bray–Curtis dissimilarity.

	df	*R* ^2^	*p*
Body condition		0.0973	0.002
Season and year	3	0.0954	0.0001
Season	2	0.0573	0.0001
Tick abundance		0.0573	0.02
Sex	1	0.0386	0.0005
Developmental stage	1	0.038	0.0001

**FIGURE 2:**
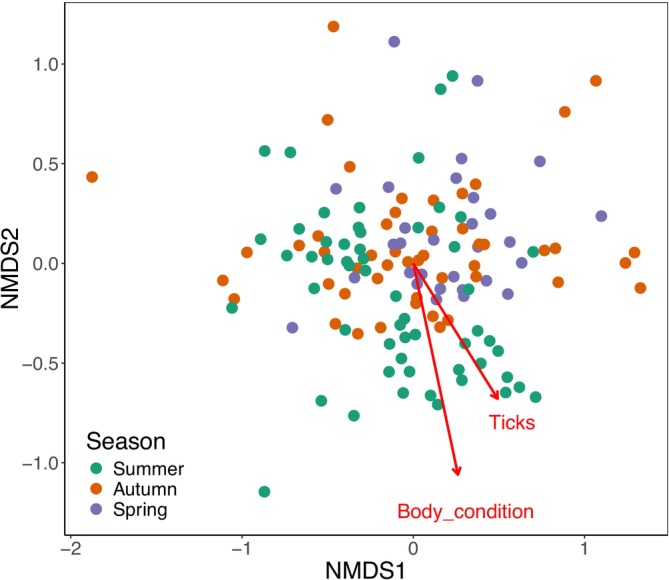
nMDS plot representing Bray–Curtis dissimilarity of the tuatara gut microbiota and variation explained by tuatara body condition and tick parasitism. Arrow length represents effect size.

Tuatara body condition was also a significant explainer of variation in the microbiota (*R* ^2^_body condition_ = 9.73, *p*_body condition_ < 0.01), as was tick load (*R* ^2^_ticks_ = 5.74, *p*_ticks_ < 0.05; Figure 2). Both explained similar amounts of variation in a similar direction. Body condition did not vary significantly with season (Figure S3; *F* = 0.29, *p* > 0.05), but tick abundance did (*F* = 7.13, *p* < 0.01), with the highest tick abundances seen in spring (Figure S2).

**FIGURE 3:**
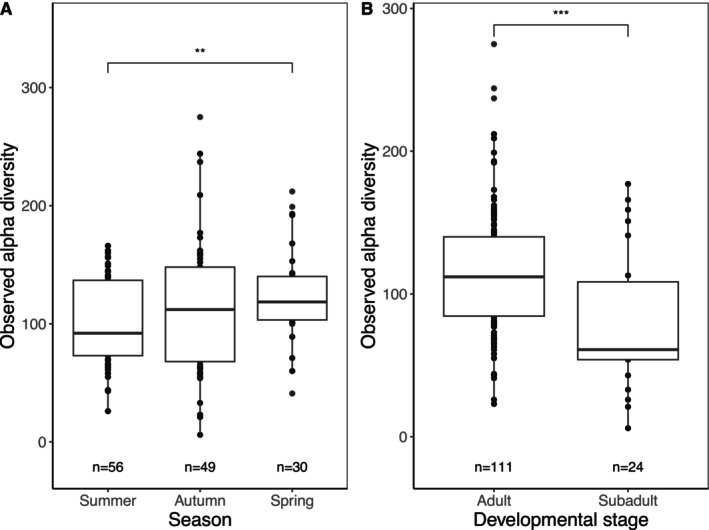
Observed alpha diversity of the tuatara gut microbiota across (A) seasons and (B) tuatara developmental stages. *p < 0.05, **p < 0.01, ***p < 0.001, ****p < 0.0001.

Bacterial alpha diversity (here represented by observed NTUs) was significantly higher in samples collected in the spring compared with those collected in the summer (*p* < 0.01; Figure 3A) but not in those collected in autumn (*p* > 0.05). Samples collected from adult tuatara also exhibited significantly higher alpha diversity than those collected from subadults (*p* < 0.001; Figure 3B), though note that the sample size for subadults was much lower than for adults. This pattern was consistent across all utilised alpha diversity metrics, with the exception of season (Figure S7).

### 3.2 Seasonal Shifts in Bacterial Community Composition

**FIGURE 4:**
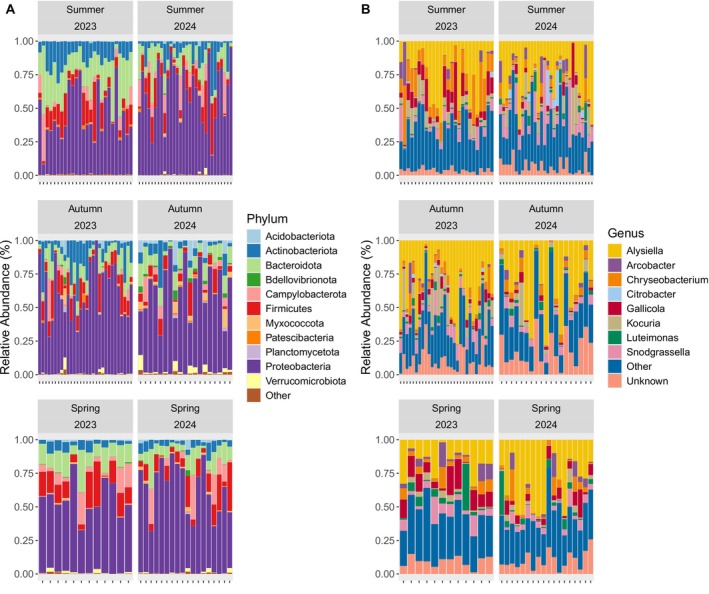
Ten most abundant bacterial (A) phyla and (B) genera within the tuatara gut microbiota across seasons.

Bacterial phyla dominating the tuatara gut included Proteobacteria (54.78% of all sequence reads), Bacteroidota (17.49%), Firmicutes (10.25%) and Actinobacteriota (9.45%), all of which were represented across seasons. Likewise, the gut was largely dominated by genera, including *Alysiella* (27.59% of reads), *Chryseobacterium* (10.05%), *Snodgrassella* (6.25%), *Gallicola* (6.07%) and *Kocuria* (5.14%), largely regardless of season or year (Figure 4). Unknown bacterial genera represented 7.62% of all reads. This dataset included 1711 NTUs across 746 unique bacterial genera.

**FIGURE 5:**
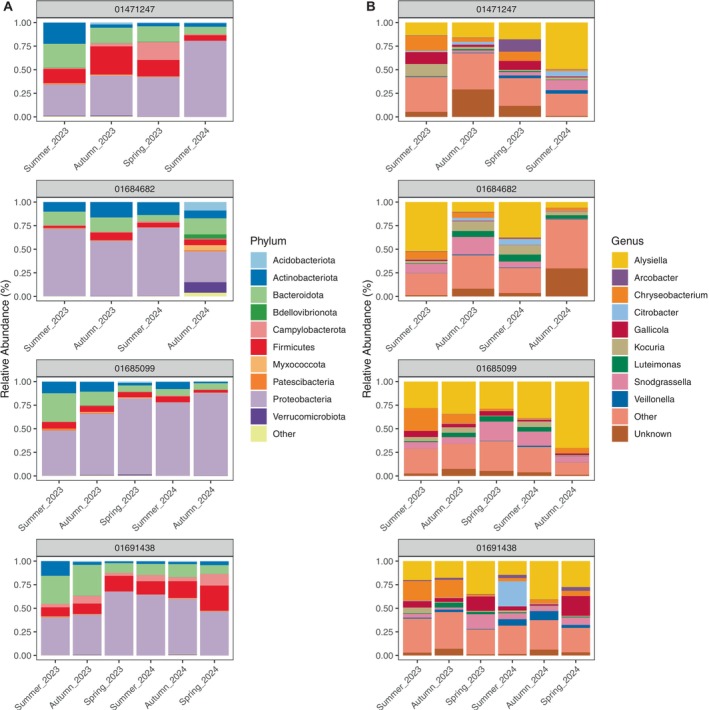
Ten most abundant bacterial (A) phyla and (B) genera across seasons in the four individual tuatara that were opportunistically sampled across multiple seasons.

Where it was possible to track the gut microbiota of individual tuatara over multiple seasons (*n* = 4), relative abundances of both phyla and genera shifted across seasons, but the presence/absence of a given taxon was largely consistent over time (Figure 5). Two exceptions to this were a large increase of *Citrobacter* in one tuatara in summer 2024, where it represented more than 25% of the community but was undetected in all other seasons (Figure 5, tuatara 01691438), and the variable relative abundance of *Chryseobacterium* in all tuatara from one season to the next (Figure 5).

**FIGURE 6:**
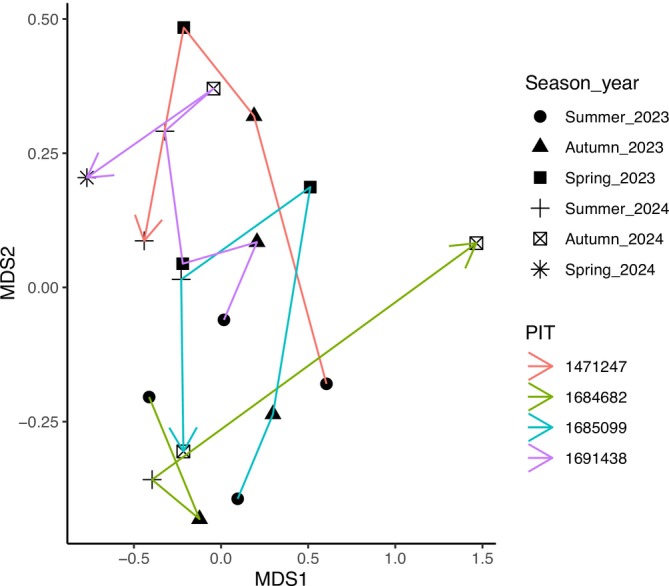
Arrow nMDS plot connecting points chronologically from summer 2023 to spring 2024 for tuatara that were opportunistically sampled in multiple seasons. PIT is the identifying mark for each individual.

Following the bacterial community of four individuals (Figure 5) over time did not reveal a consistent pattern, with no clear clustering of season or individual (Figure 6). Three tuatara (1471247, 1685099 and 1691438) showed similar patterns in the distribution of their points, though all had substantial overlap. In nMDS space, there was no consistent start or end point for the path of any tuatara, though samples were often most similar to the nearest time point (Figure 6).

**FIGURE 7:**
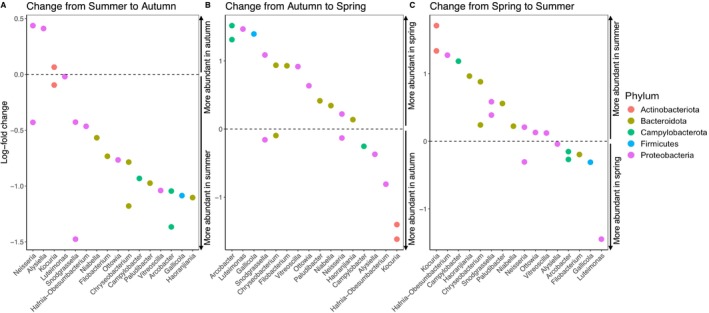
Bacterial genera with significant log‐fold change (*p* < 0.05) from (A) summer sampling to autumn sampling, (B) autumn sampling to spring sampling and (C) spring to summer sampling.

Considering the microbiota of all tuatara, a subset of bacterial genera shifted significantly both from summer to autumn and from autumn to spring (*p* < 0.05). In particular, two species of *Arcobacter* significantly decreased in autumn and significantly increased again in spring (Figure 7). Likewise, *Gallicola*, *Snodgrassella*, *Chryseobacterium*, *Filobacterium*, *Vitreoscilla*, *Ottowia* and *Paludibacter* all represented at least one NTU that decreased in abundance going into autumn but increased again in spring. *Kocuria* and *Alysiella* followed the opposite trend, increasing in abundance in autumn and decreasing in spring. *Luteimonas* increased substantially going into spring and decreased substantially again going into summer. However, none of the log‐fold changes in difference in abundance were larger than ±2, representing relatively small (albeit significant) changes in relative abundance (Figure 7). There was a core community of 19 NTUs that were found in > 80% of individual tuatara across all seasons, eight of which were from the family *Neisseriaceae* (Table S1; Figure S4). In addition to this core microbiota spanning all seasons, summer samples included three unique core members, and spring samples included seven unique core members. Many of these core members experienced significant shifts from season to season (Figure 7).

## 4 Discussion

This study examined the influence of seasonal variation on the tuatara gut microbiota, leveraging 2 years of sampling across the three active seasons for tuatara. These data allowed us to answer the following specific hypotheses: (1) that the tuatara gut microbiota varied significantly but to a limited degree between seasons, (2) that the gut microbiota did not fully reestablish in subsequent seasons and (3) that the bacterial community significantly correlated with tuatara body condition and tick abundance. This is the first seasonal analysis of the tuatara gut microbiota and furthers our understanding of the stability of the bacterial community of this animal through time and across ecologically relevant seasonal changes.

### 4.1 Sampling Period and Tuatara Body Condition Have the Strongest Link to the Gut Microbiota

As hypothesised, different seasons were associated with significantly different bacterial communities in the tuatara gut (*R* ^2^ = 5.73, *p* < 0.0001). However, season alone explained nearly half as much variation as sampling period, which included both season and year (*R* ^2^ = 9.54, *p* < 0.0001; Table 1). Though this presents evidence for seasonal shifts in the tuatara gut microbiota, it does not appear that seasonality is necessarily a cyclical process whereby the microbiota re‐establishes in subsequent seasons (Figure 1). Similarly, in other reptiles, seasonal sampling revealed significant seasonal shifts that were not necessarily re‐established in full each year (Baldo et al. 2023; Bunker et al. 2022). Though there were significant shifts in beta‐diversity related to season, there was a large degree of overlap in the genera that were present (Figure 4), with most differences being shifts in relative abundance rather than presence/absence of a bacterial genus. The significance of sampling period as a factor indicates that there are shifts in the tuatara gut over time (Table 1), though part of this may be the presence of different individuals in the dataset in different seasons. In‐depth analysis of individual tuatara over time revealed some fluctuation over time within individuals, but larger differences between individuals (Hoffbeck et al. 2024b), which is perhaps contributing to differences in sampling periods seen here.

Tuatara body condition was the strongest explainer of variation, accounting for 9.73% of variation (Table 1; Figure 2). This confirms previous findings in the tuatara gut that linked community assemblage to body condition (Hoffbeck et al. 2024a). Previous research has not supported significant shifts in tuatara body condition with seasonal variation (Hoare et al. 2006), though tuatara are known to lose weight over their least active season in winter (Cree 2014). The lowest mean weight was observed in spring (Figure S3), but this difference was not significant. Tick abundance was also correlated with shifts in the microbiota as seen previously (Hoffbeck et al. 2024a), but tick abundance also shifted significantly between seasons (Figure S2), with the highest tick load seen in spring (as has been demonstrated previously, see Godfrey et al. 2008). Ticks are largely considered not to significantly impact tuatara health. As tick load has been linked to less improvement in body condition (Godfrey et al. 2008), the correlation seen here between tick load and microbiota may be a co‐correlation between microbiota and body condition (Figure 2), or microbiota and season (Figure 1). Parasitism can drive microbiota‐mediated immune response in some species (Zheng et al. 2020), but its role in the tuatara gut will require further investigation.

Tuatara sex and developmental stage were also significant explainers of variation in the gut microbiota (*R* ^2^_age_ = 3.80, *p*_age_ < 0.0001; *R* ^2^_sex_ = 3.86, *p*_sex_ < 0.001), though with substantial overlap (Figure 1). Male and female tuatara did not differ significantly in alpha diversity, though subadult tuatara demonstrated significantly lower alpha diversity than adults (*p* < 0.001). This difference mirrors findings in many species (Martino et al. 2022; Xu and Zhang 2021), though reptiles are underrepresented in this literature. Though alpha diversity was lower in subadults, they did display greater spread in beta‐diversity (Figure 1). Differences in microbiota diversity between males and females have been demonstrated in other reptile species (Bunker et al. 2022), and in tuatara previously (Hoffbeck et al. 2024a), but considering beta‐diversity and taxonomy it is clear that tuatara have a fairly consistent gut community across males and females, and between subadults and adults (Figure 1).

### 4.2 Taxonomy of the Tuatara Gut Across Seasons Was Generally Consistent

Across all seasons, tuatara demonstrated a core community consisting of 19 NTUs from 15 bacterial genera. A further 13 NTUs from 12 genera varied seasonally, with spring having the largest core (Table S1). This is similar to the core reported for other reptiles and amphibians (Alemany et al. 2022; Bunker et al. 2022; Douglas et al. 2021), and largely consisted of the most dominant bacteria. In particular, *Alysiella* (family *Neisseriaceae*, phylum Proteobacteria) accounted for nearly a third of all reads and was present in virtually every sample (Figure 4). Other members of family *Neisseriaceae* also dominated the gut, including *Snodgrassella*, *Kingella* and *Neisseria* (Table S1). Family *Neisseriaceae* was the overall dominant family in the majority of samples, representing 38.8% of all reads (Figure S4). This family contains both aerobic and facultatively anaerobic members (Dewhirst et al. 1989), most commonly associated with either mammalian oral cavities (Nyongesa et al. 2022) or, as in the case of its most infamous member *Neisseria gonorrhoeae* , in the urogenital tract (Quillin and Seifert 2018).

*Neisseriaceae* has not been commonly described in reptiles, and the particular genera found in tuatara are not usual members of the gut microbiota of class Reptilia (Hoffbeck et al. 2023; Huang et al. 2022). The genus *Alysiella*, first cultured from sheep saliva, has only rarely been described (Nyongesa et al. 2022; Xie and Yokota 2004) and includes only two species ( *A. filiformis* and *A. crassis*), both of which were represented in this dataset. However, a potentially novel species of *Alysiella* was the only NTU that qualified for the core and that made up the vast majority of *Alysiella* present in the tuatara gut. Both described species of *Alysiella* demonstrate glucose, maltose, mannitol and sucrose metabolism, as well as gelatin and casein hydrolysis (Xie and Yokota 2004), but the specific role that this genus is playing in the tuatara gut will require future research. Tuatara have a notable lack of genus *Bacteroides*, one of the primary players in carbohydrate metabolism in other vertebrate guts (Shin et al. 2024; Wexler and Goodman 2017; Wexler 2007) and in reptile guts specifically (Hoffbeck et al. 2023, 2025). Investigation of the specific metabolic role that genus *Alysiella* and family *Neisseriaceae* play for tuatara may help reveal what role the bacterial community serves for this host and if *Alysiella* contributes similar functionality that *Bacteroides* serves for other hosts.

Individual‐level variation in tuatara sampled opportunistically across multiple seasons revealed general consistency, though with shifts in relative abundance between seasons (Figure 5). Though individuals did exhibit some differences, visualisation of these individuals with nMDS did not reveal any clustering by individual or season (*p* > 0.05; Figure 6). Individual‐level trends were apparent in some previous investigations of tuatara (Hoffbeck et al. 2024b), but the core and dominant bacteria in tuatara appear to be largely consistent across individuals, albeit with some differences in relative abundance. Though only a single individual was sampled in all six seasons, precluding statistical analysis, this individual did not demonstrate any clear cyclic trends in beta diversity (Figures 5 and 6).

### 4.3 Season Significantly Shifts Tuatara Microbiota

Though large‐scale shifts in gut microbiota were not observed between seasons, there were significant shifts in alpha‐ and beta‐diversity, with specific genera increasing or decreasing from season to season. Most, but not all, bacterial genera that significantly changed in abundance between seasons were core community members. Both NTUs associated with *Kocuria* showed strong seasonal shifts: *Kocuria* decreased nearly two‐fold from autumn to spring and increased comparably from spring to summer (Figure 7). *Arcobacter* also changed cyclically, going from low abundance in autumn to higher abundance in spring, shifting back to lower abundance in summer. Though present in the core throughout seasons, *Luteimonas* was also significantly more abundant in spring than in summer or autumn (Figure 7). In other reptiles, mucin‐utilising and fermentative bacteria, including *Bacteroides*, were significantly more abundant in winter during the inactive period (Baldo et al. 2023; Tang et al. 2019). The increased core community identified in spring (our proxy for the less active winter period) may indicate a specialised community for this shift in access to nutrients (Tang et al. 2019; Weng et al. 2016).

Significantly higher alpha diversity was displayed in spring (*p* < 0.01) compared with summer (Figure 3). Spring is when tuatara are re‐entering their active period and is the laying season for female tuatara carrying eggs (Cree 2014). Mating season, and therefore the most social period for tuatara, occurs in summer. Some squamate reptiles demonstrate the same trend of lowest diversity during the period of the highest sociality (Bunker et al. 2022), but most other studies of seasonality on ectothermic species link seasonal shifts to changing dietary availability (Gao et al. 2023; Huang and Liao 2021; Park and Do 2024; Tang et al. 2019). Though New Zealand does experience seasonal shifts which affect plant life, tuatara are largely insectivorous (Fraser 2012), a food source that is available year‐round to tuatara (though consumption of seabirds and berries—both seasonally available—should be considered as well, see Lamar et al. 2022). Additionally, though there is some evidence of dietary items contributing bacteria to the tuatara gut, the extent of this contribution was weak (Hoffbeck et al. 2024b). A combination of the lack of seasonality in the availability of primary dietary items and the low influence of dietary items on the overall gut community may both contribute to the relative stability of the tuatara gut over seasons.

### 4.4 Concluding Remarks

This work represents the first long‐term investigation of the tuatara gut microbiota encompassing seasonal variation and included periods associated with mating, egg‐laying and return to activity. We identify a bacterial community consistent with tuatara across sites in New Zealand and in captivity, and consistent over time, further indicating the long‐term stability of the tuatara gut. We found significant links to body condition and tick load, both of which have implications for how the microbiota interacts with tuatara health. We also demonstrate significant shifts in the tuatara gut associated with season and identify specific genera that change cyclically across seasons. Despite these differences, the dominant and core bacteria in the tuatara gut remained remarkably stable over time and across individuals. Here, we provide further insight into the unique gut community of this taonga (treasured) species and utilise a large dataset to investigate both broad‐scale and specific trends in the gut over time. This work again sets tuatara apart from their closest reptiles and provides more context for the gut community of this ancient species, with implications for the role of the gut microbiota in tuatara health and ecology.

## Author Contributions

**Carmen Hoffbeck:** conceptualization (equal), data curation (lead), formal analysis (lead), funding acquisition (lead), investigation (equal), methodology (equal), project administration (lead), visualization (lead), writing – original draft (lead), writing – review and editing (equal). **Danielle M. R. L. Middleton:** conceptualization (equal), funding acquisition (equal), supervision (equal), writing – review and editing (equal). **Nicola J. Nelson:** conceptualization (equal), investigation (equal), methodology (equal), project administration (equal), resources (equal), supervision (equal), writing – review and editing (equal). **Michael W. Taylor:** conceptualization (equal), supervision (equal), writing – review and editing (equal).

## Conflicts of Interest

The authors declare no conflicts of interest.

## Supporting information


Data S1.

## Data Availability

The datasets presented in this study can be found in online repositories. The names of the repository/repositories and accession number(s) can be found at: https://www.ncbi.nlm.nih.gov/genbank/, PRJNA1199060. The workflow for analysis and visualisation in R is available in the supplement.
