# Supplementary material for: Season, Body Condition and Developmental Stage Influence the Gut Microbiota of the Sole Living Rhynchocephalian Reptile (Sphenodon punctatus)
Source: Ecol Evol. 2025 Apr 11;15(4):e71068. doi: 10.1002/ece3.71068 (PMC11992361; doi:10.1002/ece3.71068)
Supplement: Supplementary file 1 — Data S1. [file ECE3-15-e71068-s001.zip › ECE371068-sup-0004-Supplement.docx]

**Season, body condition, and developmental stage influence the gut microbiota of the sole living rhynchocephalian reptile (*Sphenodon punctatus*)**

Hoffbeck, Carmen^1^; Middleton, Danielle MRL^2^; Nelson, Nicola J^3^; Taylor, Michael W^4,*^

^1^School of Biological Sciences, University of Auckland, Auckland, New Zealand

^2^Manaaki Whenua - Landcare Research, Lincoln, New Zealand

^3^School of Biological Sciences, Victoria University of Wellington, Wellington, New Zealand

^*^Corresponding author: Mike Taylor, mw.taylor@auckland.ac.nz

**Supplemental tables and figures**

Figure S1. Rarefaction curve for all samples. Samples were rarefied to 1000 reads each.

Figure S2. Average tick abundance (±SE) per tuatara in each season. *p < .05, **p < .01, ***p < .001, ****p < .0001. Box plots indicate median ± SE and black dots indicate outliers (datapoints ±1.5x the interquartile range), and black lines indicate minimum and maximum values that are not outliers.

Figure S3. Average tuatara weight (±SE) in each season. No differences were significant between seasons. Box plots indicate median ± SE and black dots indicate outliers (datapoints ±1.5x the interquartile range), and black lines indicate minimum and maximum values that are not outliers.


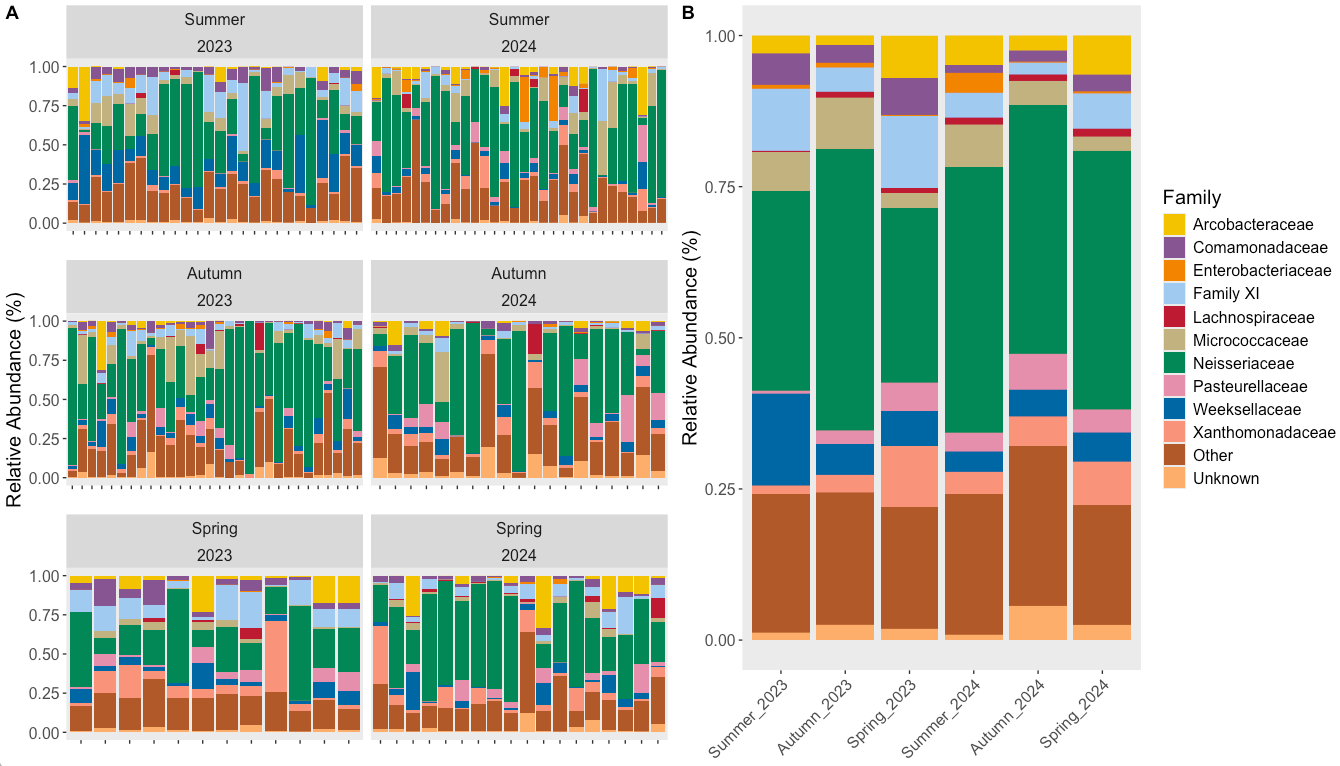


Figure S4. Top 10 bacterial families across sampling periods. (A) Individual-level variation in family composition, and (B) overall variation in family composition.


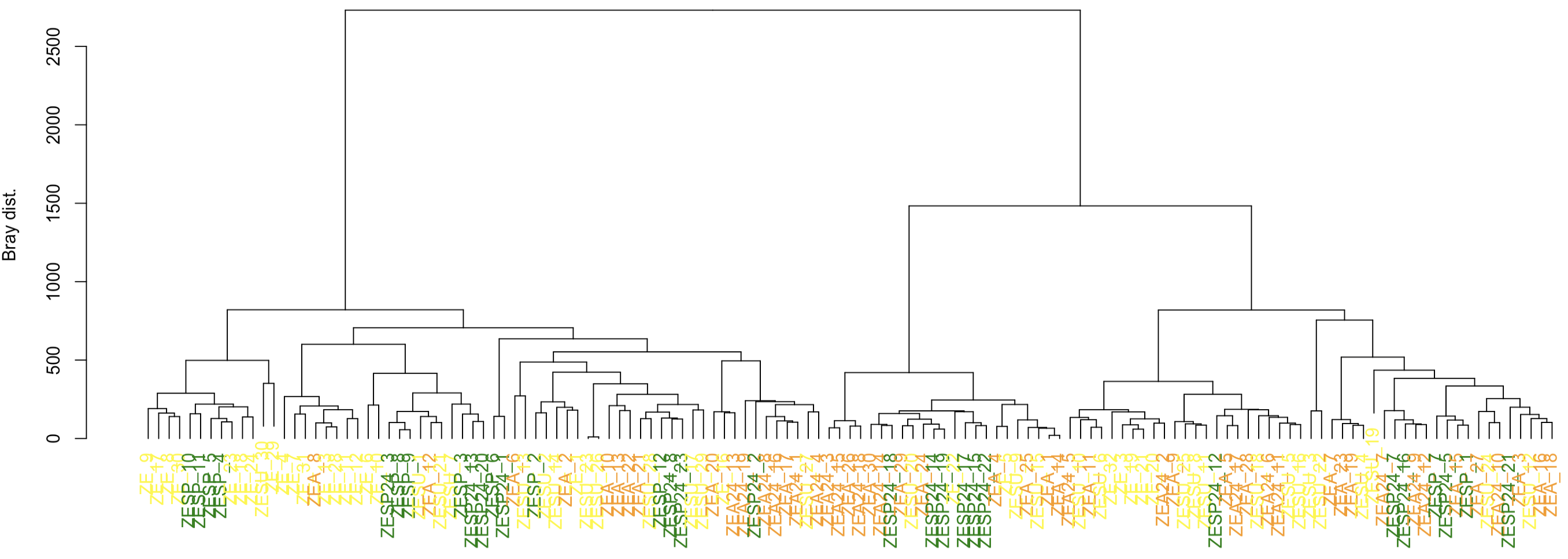


**B**

**A**


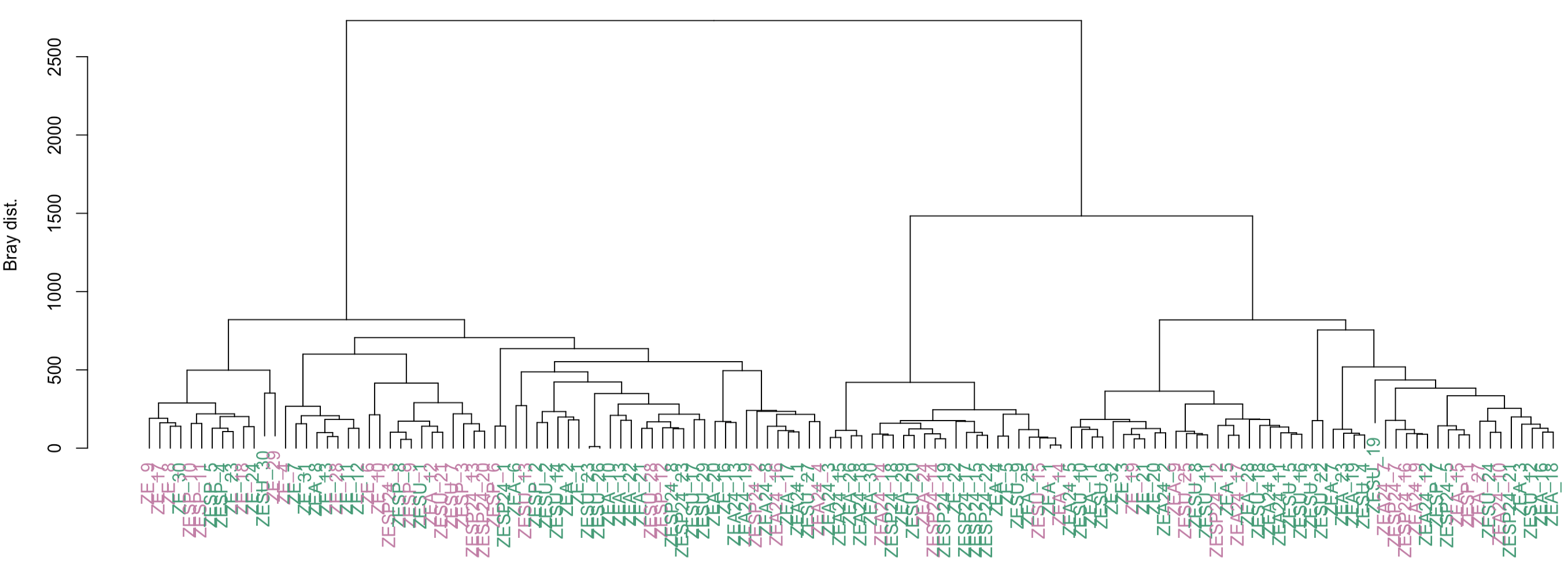


**C**


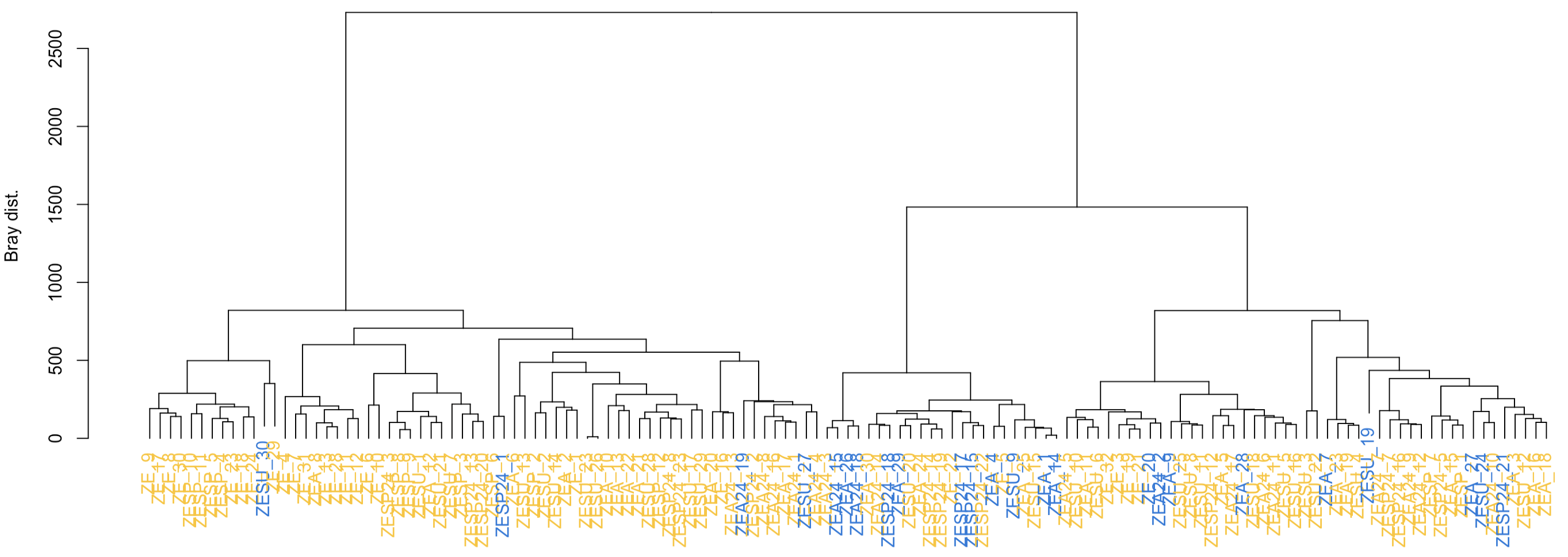


Figure S5. Ward hierarchical clustering based on Bray-curtis dissimilarity between samples. Samples are coloured by (A) season, (B) developmental stage, and (C) sex.

Figure S6. Number of shared and specific taxa grouped by (A) season, (B) tuatara developmental stage, and (C) tuatara sex.

Figure S7. Alpha-diversity of the tuatara gut microbiota calculated using Shannon (A-C), Simpson (D-F), and Chao1 (G-I) diversity metrics.

Table S1. Core community members in summer, autumn, and spring. The eight members of family *Neisseriaceae* are included first.

| Summer – 24 members | Autumn – 21 members | Spring – 28 members |
| --- | --- | --- |
| *Alysiella* sp. | *Alysiella* sp. | *Alysiella* sp. |
| *Kingella* sp. | *Kingella* sp. | *Kingella* sp. |
| *Kingella kingae* | *Kingella kingae* | *Kingella kingae* |
| *Snodgrassella* sp. | *Snodgrassella* sp. | *Snodgrassella* sp. |
| *Snodgrassella alvi* | *Snodgrassella alvi* | *Snodgrassella alvi* |
| *Neisseria* sp. | *Neisseria* sp. | *Neisseria* sp. |
| *Neisseria weaveri* | *Neisseria weaveri* | *Neisseria weaveri* |
| *Vitreoscilla* sp. | *Vitreoscilla* sp. | *Vitreoscilla* sp. |
| *Niabella* sp. | *Niabella* sp. | *Niabella* sp. |
| *Gallicola* sp. | *Gallicola* sp. | *Gallicola* sp. |
| *Chryseobacterium* sp. | *Chryseobacterium* sp. | *Chryseobacterium* sp. |
| *Kocuria* sp. | *Kocuria* sp. | *Kocuria* sp. |
| *Kocuria carniphila* | *Kocuria carniphila* | *Kocuria carniphila* |
| *Luteimonas* sp. | *Luteimonas* sp. | *Luteimonas* sp. |
| *Paludibacter* sp. | *Paludibacter* sp. | *Paludibacter* sp. |
| *Propioniciclava* sp. | *Propioniciclava* sp. | *Propioniciclava* sp. |
| *Ferruginibacter* sp. | *Ferruginibacter* sp. | *Ferruginibacter* sp. |
| *Flavisolibacter ginsenosidimutans* | *Flavisolibacter ginsenosidimutans* | *Flavisolibacter ginsenosidimutans* |
| *Haoranjiania* sp. | *Haoranjiania* sp. | *Haoranjiania* sp. |
| *Ottowia* sp. |  | *Ottowia* sp. |
|  | *Stenotrophomonas* sp. | *Stenotrophomonas* sp. |
| *Corynebacterium* sp. | *Corynebacterium* sp. |  |
| *Antricoccus* sp. |  |  |
| *Kocuria rhizophila* |  |  |
| *Hafnia-Obesumbacterium alvei* |  |  |
|  |  | *Tessaroccus flavus* |
|  |  | *Comamonadaceae* sp. |
|  |  | *Conexibacter* sp. |
|  |  | *Lysobacter* sp. |
|  |  | *Comamonas* sp. |
|  |  | *Pseudomonas* sp. |
|  |  | *Paracoccus* sp. |

**Supplemental code for processing and analysis**

1. Trim reads with Nanofilt

module load nanofilt/2.6.0-gimkl-2020a-Python-3.8.2

for i in *.fastq;

do

NanoFilt -q 10 -l 1500 ${i} > trimmed_${i}

done

1. Process reads with Emu

module load Miniconda3

source activate emu

for i in *.fastq;

do

emu abundance ${i} --db silva_database --keep-counts

done

emu combine-outputs results/ tax_id –counts

1. Analysis in R

### tuatara gut microbiome: seasonal analysis ###

# clear workspace

rm(list = ls())

# load libraries

library(decontam)

library(phyloseq)

library(tidyverse)

library(vegan)

library(SRS)

library(cowplot)

library(ggsignif)

library(ggpubr)

library(EnvStats)

library(microbiome)

library(edgeR)

## load data ####

count_tab <- read.table("seasonal-count-tab.txt", header=T, row.names=1, check.names=F, sep="\t")

tax_tab <- as.matrix(read.table("seasonal_tax_tab.txt", header=T, row.names=1, check.names=F, sep="\t"))

sample_info_tab <- read.table("seasonal-meta-data.txt", quote = "", header=T, row.names=1, check.names=F, sep="\t")

rownames(sample_info_tab) <- sample_info_tab$ID

## decontam ####

vector_for_decontam <- c(rep(FALSE, 30), rep(TRUE, 1),rep(FALSE,12), rep(TRUE,1), rep(FALSE,30), rep(TRUE,18),rep(FALSE,51),rep(TRUE,2),rep(FALSE,23),rep(TRUE,1))

contam_df <- isContaminant(t(count_tab), neg=vector_for_decontam)

table(contam_df$contaminant)

contam_asvs <- row.names(contam_df[contam_df$contaminant == TRUE, ])

tax_tab[row.names(tax_tab) %in% contam_asvs, ]

asv_tab_no_contam <- count_tab[!row.names(count_tab) %in% contam_asvs, ]

asv_tax_no_contam <- tax_tab[!row.names(tax_tab) %in% contam_asvs, ]

write.table(asv_tab_no_contam, "ASVs_counts-no-contam.tsv", sep="\t", quote=F, col.names=NA)

write.table(asv_tax_no_contam, "ASVs_taxonomy-no-contam.tsv", sep="\t", quote=F, col.names=NA)

### make a phyloseq object and remove blanks ####

count_tab <- read.table("ASVs_counts-no-contam.tsv", header=T, row.names=1, check.names=F, sep="\t")[ , -c(31,44,75:92,144:145,169)]

tax_tab <- as.matrix(read.table("ASVs_taxonomy-no-contam.tsv", header=T, row.names=1, check.names=F, sep="\t"))

sample_info_tab <- read.table("seasonal-meta-data-SC412444.txt", quote = "", header=T, row.names=1, check.names=F, sep="\t")

rownames(sample_info_tab) <- sample_info_tab$ID

count_tab_phy <- otu_table(count_tab, taxa_are_rows=T)

tax_tab_phy <- tax_table(tax_tab)

sample_info_tab_phy <- sample_data(sample_info_tab)

ASV_physeq <- phyloseq(count_tab_phy, tax_tab_phy, sample_info_tab_phy)

### rarifaction curve ####

rarecurve(t(count_tab), step=100, lwd=2, ylab="ASVs", label=F,xlim=c(0, 2000))

abline(v=1000)

count_tab_r <- SRS(count_tab, Cmin = 1000, set_seed = TRUE)

count_tab_r <- as.data.frame(count_tab_r)

rows <- rownames(count_tab)

rownames(count_tab_r) <- rows

colnames(count_tab_r)

common_names <- intersect(colnames(count_tab_r), rownames(sample_info_tab))

sample_info_tab_r <- sample_info_tab[common_names,]

r_count_phy <- otu_table(count_tab_r, taxa_are_rows=T)

sample_info_tab_phy <- sample_data(sample_info_tab_r)

r_tax_phy <- tax_table(tax_tab)

r_physeq <- phyloseq(r_count_phy, r_tax_phy, sample_info_tab_phy)

adult_physeq_r <- subset_samples(r_physeq,Age=="Adult")

r_count_adult <- as.data.frame(as(otu_table(adult_physeq_r), "matrix"))

r_data_adult <- as.data.frame(as(sample_data(adult_physeq_r), "matrix"))

### ordinate ####

ord <- ordinate(r_physeq, method = "NMDS", distance = "bray", trymax = 100)

ord1 <- ordinate(adult_physeq_r, method = "NMDS", distance = "bray", trymax = 100)

stressplot(ord)

season_o<-plot_ordination(r_physeq, ord, color = "Season_year") + geom_point(size = 2) +

scale_color_manual(values=c("#1B9E77","#D95F02","#7570B3","#66A61E","#E6AB02","#E7298A")) + stat_ellipse(type = "norm", linetype = 2) +

theme_bw()+ theme(text = element_text(size = 15)) +

theme(panel.grid.major = element_blank(), panel.grid.minor = element_blank(),

panel.background = element_blank(), axis.line = element_line(colour = "black"))

age_o<-plot_ordination(r_physeq, ord, color = "Age") +

geom_point(size = 2) + scale_color_manual(values=c("#FFC20A","#0C7BDC")) +

stat_ellipse(type = "norm", linetype = 2) +

theme_bw()+ theme(text = element_text(size = 15)) +

theme(panel.grid.major = element_blank(), panel.grid.minor = element_blank(),

panel.background = element_blank(), axis.line = element_line(colour = "black"))

sex_o<-plot_ordination(adult_physeq_r, ord1, color = "Sex") +

geom_point(size = 2) + scale_color_manual(values=c("#CC79A7","#009E73")) +

stat_ellipse(type = "norm", linetype = 2) +

theme_bw()+

theme(text = element_text(size = 15)) +

theme(panel.grid.major = element_blank(), panel.grid.minor = element_blank(),

panel.background = element_blank(), axis.line = element_line(colour = "black"))

plot_grid(season_o,age_o,sex_o,nrow=1,labels=c("A","B","C"))

# discrete variables permanova

r_permanova <- adonis2(t(count_tab_r) ~ Season*Season_year*Age,

data = sample_info_tab_r, permutations=9999, method = "bray")

r_permanova_adult <- adonis2(t(r_count_adult) ~ Season*Sex,

data = r_data_adult, permutations=9999, method = "bray")

dist_bray <- phyloseq::distance(r_physeq, method = "bray")

beta <- betadisper(dist_bray, sample_info_tab_r$Age)

permutest(beta)

beta <- betadisper(dist_bray, sample_info_tab_r$Sex)

permutest(beta)

beta <- betadisper(dist_bray, sample_info_tab_r$Season)

permutest(beta)

beta <- betadisper(dist_bray, sample_info_tab_r$Season_year)

permutest(beta)

# continuous variables permanova

manova <- metaMDS(t(count_tab_r),distance="bray",k=2,engine=c("monoMDS"))

df <- tibble::rownames_to_column(sample_info_tab_r, "ID2")

envfit <- envfit(manova ~ Body_condition+Ticks, data = df, permutations = 9999, na.rm=TRUE)

ordiplot(manova, type = "n")

orditorp(manova, display = "sites", col = "black", cex = 1)

plot(envfit, col = "goldenrod", cex = 1)

envfit.df <- as.data.frame(scores(envfit, "vectors"))

envfit.df$variable <- rownames(envfit.df)

season_o<-plot_ordination(r_physeq, ord, color = "Season") +

geom_point(size = 4) +

stat_ellipse(type = "norm", linetype = 2) +

theme_bw()+ theme(text = element_text(size = 20)) +

scale_color_manual(values=c("#1B9E77","#D95F02","#7570B3","#E7298A","#66A61E","#E6AB02")) + theme(panel.grid.major = element_blank(), panel.grid.minor = element_blank(),

panel.background = element_blank(), axis.line = element_line(colour = "black"))

season_e<-plot_ordination(r_physeq, ord, color = "Season") +

geom_point(size = 4) +

theme_bw()+ theme(text = element_text(size = 20)) +

scale_color_manual(values=c("#1B9E77","#D95F02","#7570B3","#E7298A","#66A61E","#E6AB02"))+ theme(panel.grid.major = element_blank(), panel.grid.minor = element_blank(),

panel.background = element_blank(), axis.line = element_line(colour = "black"))

arrow_plot <- season_e +

geom_segment(data=envfit.df,

aes(x=0, xend=NMDS1*3.5, y=0, yend = NMDS2*3.5),

arrow = arrow(length=unit(0.25,"cm")),

colour="red", size=1,

inherit.aes = FALSE) +

geom_text(data = envfit.df,

aes(x=NMDS1*4.1, y = NMDS2*4.1,label=variable),

size=6,color="red", inherit.aes = FALSE, check_overlap = TRUE)+

theme(panel.grid.major = element_blank(), panel.grid.minor = element_blank(),

panel.background = element_blank(), axis.line = element_line(colour = "black"))

plot_grid(season_e,arrow_plot,ncol=2)

### arrow plot ####

info_pit <- sample_info_tab_r %>%

rownames_to_column('samps') %>%

group_by(PIT) %>% filter(n()>3) %>% filter(PIT!="None") %>%

column_to_rownames('samps')

col_extracted <- intersect(rownames(info_pit), colnames(count_tab_r))

count_pit <- count_tab_r[,col_extracted]

count_tab_pit <- otu_table(count_pit, taxa_are_rows=T)

tax_tab_phy <- tax_table(tax_tab)

sample_info_tab_pit <- sample_data(info_pit)

r_pit_phy <- phyloseq(count_tab_pit, tax_tab_phy, sample_info_tab_pit)

r_pit_phy <- subset_samples(r_pit_phy,ID != "ZEA24_4")

r_pit_phy <- subset_samples(r_pit_phy, PIT!="1172455")

ord_pit <- ordinate(r_pit_phy, method = "NMDS", distance = "bray", trymax = 100)

info_pit2 <- info_pit %>% rownames_to_column('Sample')

nmds <- as.data.frame(ord_pit$points) %>% rownames_to_column('Sample')

nmds <- nmds |>

left_join(info_pit2)

nmds %>%

arrange(Season_year) %>%

ggplot(aes(x = MDS1, y = MDS2, shape=Season_year)) +

geom_point(size=3) +

geom_path(aes(x = MDS1, y = MDS2, group = PIT, color=PIT),

arrow = arrow(length = unit(0.55, "cm"))) +

theme(panel.grid.major = element_blank(), panel.grid.minor = element_blank(),

panel.background = element_blank(), axis.line = element_line(colour = "black"))

### alpha diversity ####

my_comparisons <- list(c("Summer","Autumn"),c("Summer","Spring"), c("Spring","Autumn"))

symnum.args = list(cutpoints = c(0, 0.0001, 0.001, 0.01, 0.05, 1), symbols = c("****", "***", "**", "*"))

season_a <- plot_richness(r_physeq, x="Season", measures=c("Observed")) +

geom_boxplot() +stat_n_text() +

theme_bw() + theme(legend.title = element_blank(),legend.position="none",strip.background = element_blank(),

strip.text.x = element_blank()) + xlab("Season") + ylab("Observed alpha diversity") +

stat_compare_means(method = "wilcox.test", comparisons = my_comparisons, label = "p.signif", symnum.args = symnum.args)+

theme(text = element_text(size = 15)) +

theme(panel.grid.major = element_blank(), panel.grid.minor = element_blank(),

panel.background = element_blank(), axis.line = element_line(colour = "black"))

my_comparisons <- list(c("Subadult","Adult"))

symnum.args = list(cutpoints = c(0, 0.0001, 0.001, 0.01, 0.05, 1), symbols = c("****", "***", "**", "*"))

age_a <- plot_richness(r_physeq, x="Age", measures=c("Observed")) +

geom_boxplot() +stat_n_text() +

theme_bw() + theme(legend.title = element_blank(),legend.position="none",strip.background = element_blank(),

strip.text.x = element_blank()) + xlab("Age") + ylab("Observed alpha diversity") +

stat_compare_means(method = "wilcox.test", comparisons = my_comparisons, label = "p.signif", symnum.args = symnum.args)+

theme(text = element_text(size = 15)) +

theme(panel.grid.major = element_blank(), panel.grid.minor = element_blank(),

panel.background = element_blank(), axis.line = element_line(colour = "black"))

my_comparisons <- list(c("Male","Female"))

symnum.args = list(cutpoints = c(0, 0.0001, 0.001, 0.01, 0.05, 1), symbols = c("****", "***", "**", "*"))

sex_a <- plot_richness(adult_physeq_r, x="Sex", measures=c("Observed")) +

geom_boxplot() +stat_n_text() +

theme_bw() + theme(legend.title = element_blank(),legend.position="none",strip.background = element_blank(),

strip.text.x = element_blank()) + xlab("Sex") + ylab("Observed alpha diversity") +

stat_compare_means(method = "wilcox.test", comparisons = my_comparisons, label = "p.signif", symnum.args = symnum.args)+

theme(text = element_text(size = 15)) +

theme(panel.grid.major = element_blank(), panel.grid.minor = element_blank(),

panel.background = element_blank(), axis.line = element_line(colour = "black"))

plot_grid(season_a,age_a,nrow=1,labels=c("A","B"))

### taxonomy plots ####

my_colors <- c("#F3C300","#875692" ,"#F38400" ,"#A1CAF1" ,"#BE0032", "#C2B280", "#008856", "#E68FAC", "#0067A5","#F99379","#B15928","#FDAE6B","#604E97")

phy2 <- r_physeq %>% aggregate_rare(level = "phylum", detection=2000/100,prevalence = 1/100) %>% microbiome::transform(transform = "compositional")

fam2 <- r_physeq %>% aggregate_rare(level = "family", detection=16000/100,prevalence = 1/100) %>%microbiome::transform(transform = "compositional")

gen2 <- r_physeq %>% aggregate_rare(level = "genus", detection=16000/100,prevalence = 1/100) %>%microbiome::transform(transform = "compositional")

phy_melt <- psmelt(phy2)

fam_melt <- psmelt(fam2)

gen_melt <- psmelt(gen2)

phy <- ggplot(phy_melt, aes(x = Sample, y = Abundance, fill = factor(phylum, levels=c("Acidobacteriota","Actinobacteriota","Bacteroidota","Bdellovibrionota","Campylobacterota","Firmicutes","Myxococcota","Patescibacteria","Planctomycetota","Proteobacteria","Verrucomicrobiota","Other","Unknown")))) +

geom_bar(aes(),stat="identity",position="fill") +theme(text = element_text(size = 15)) +

ylab("Relative Abundance (%)") + facet_wrap(~Season+Year,scales = "free_x", drop = F, ncol= 2) +

theme(panel.grid.major = element_blank(), panel.grid.minor = element_blank()) +

scale_fill_brewer(palette="Paired") + guides(fill=guide_legend(title="Phylum")) +

theme(axis.title.x=element_blank()) +

theme(axis.text.x=element_text(colour="white"))

gen <- ggplot(gen_melt, aes(x = Sample, y = Abundance, fill=factor(genus,levels=c("Alysiella","Arcobacter","Chryseobacterium","Citrobacter","Gallicola","Hafnia- Obesumbacterium","Kocuria","Luteimonas", "Ottowia","Snodgrassella", "Other", "Unknown")))) +

geom_bar(aes(),stat="identity",position="fill") +theme(text = element_text(size = 15)) +

ylab("Relative Abundance (%)") + facet_wrap(~Season+Year,scales = "free_x", drop = F, ncol= 2) + theme(axis.title.x=element_blank()) +

theme(panel.grid.major = element_blank(), panel.grid.minor = element_blank()) +

scale_fill_manual(values=my_colors) + guides(fill=guide_legend(title="Genus")) +

theme(axis.text.x=element_text(colour="white"))

plot_grid(phy,gen,axis="tblr",align="hv")

fam <- ggplot(fam_melt, aes(x = Sample, y = Abundance, fill=factor(family,levels=c("Arcobacteraceae","Comamonadaceae","Enterobacteriaceae"," Family XI", "Lachnospiraceae","Micrococcaceae","Neisseriaceae","Pasteurellaceae", "Weeksellaceae","Xanthomonadaceae","Other","Unknown")))) +

geom_bar(aes(),stat="identity",position="fill") +theme(text = element_text(size = 15)) +

ylab("Relative Abundance (%)") + facet_wrap(~Season+Year,scales = "free_x", drop = F, ncol= 2) +

theme(panel.grid.major = element_blank(), panel.grid.minor = element_blank()) +

scale_fill_manual(values=my_colors) + guides(fill="none") +

theme(axis.title.x=element_blank()) +

theme(axis.text.x=element_text(colour="white"))

fam2 <- ggplot(fam_melt, aes(x = Season_year, y = Abundance, fill=factor(family,levels=c("Arcobacteraceae","Comamonadaceae","Enterobacteriaceae", "Family XI","Lachnospiraceae","Micrococcaceae","Neisseriaceae","Pasteurellaceae", "Weeksellaceae","Xanthomonadaceae","Other","Unknown")))) +

geom_bar(aes(),stat="identity",position="fill") +theme(text = element_text(size = 15)) +

ylab("Relative Abundance (%)") +

theme(panel.grid.major = element_blank(), panel.grid.minor = element_blank()) +

scale_fill_manual(values=my_colors) + guides(fill=guide_legend(title="Family")) +

theme(axis.title.x=element_blank())+ theme(axis.text.x = element_text(angle = 45, vjust = 1, hjust=1))

plot_grid(fam, fam2, labels=c("A","B"))

### individual change ####

info_pit <- sample_info_tab_r %>%

rownames_to_column('samps') %>%

group_by(PIT) %>% filter(n()>3) %>% filter(PIT!="None") %>%

column_to_rownames('samps')

col_extracted <- intersect(rownames(info_pit), colnames(count_tab_r))

count_pit <- count_tab_r[,col_extracted]

count_tab_pit <- otu_table(count_pit, taxa_are_rows=T)

tax_tab_phy <- tax_table(tax_tab)

sample_info_tab_pit <- sample_data(info_pit)

r_pit_phy <- phyloseq(count_tab_pit, tax_tab_phy, sample_info_tab_pit)

ind_physeq <- subset_samples(r_physeq, PIT=="1691438"|PIT=="1684682"|PIT=="1685099"|PIT=="1471247")

my_colors <- c("#F3C300","#875692" ,"#F38400" ,"#A1CAF1" ,"#BE0032", "#C2B280", "#008856", "#E68FAC", "#0067A5","#F99379","#B15928","#FDAE6B","#604E97")

phy3 <- ind_physeq %>% aggregate_rare(level = "phylum", detection=1000/100,prevalence = 1/100) %>% microbiome::transform(transform = "compositional")

gen3 <- ind_physeq %>% aggregate_rare(level = "genus", detection=7000/100,prevalence = 1/100) %>% microbiome::transform(transform = "compositional")

phy_melt_ind <- psmelt(phy3)

gen_melt_ind <- psmelt(gen3)

phy_ind <- ggplot(phy_melt_ind, aes(x = Season_year, y = Abundance, fill = factor(phylum, levels=c("Acidobacteriota","Actinobacteriota","Bacteroidota","Bdellovibrionota","Campylobacterota","Firmicutes","Myxococcota","Patescibacteria","Planctomycetota","Proteobacteria","Verrucomicrobiota","Other","Unknown")))) + geom_bar(aes(),stat="identity",position="fill") +

ylab("Relative Abundance (%)") + facet_wrap(~PIT,scales = "free_x", drop = F, ncol= 1) +

theme(panel.grid.major = element_blank(), panel.grid.minor = element_blank()) +

scale_fill_brewer(palette="Paired") + guides(fill=guide_legend(title="Phylum")) +

theme(axis.title.x=element_blank()) + theme(axis.text.x = element_text(angle = 45, vjust = 1, hjust=1))

gen_ind <- ggplot(gen_melt_ind, aes(x = Season_year, y = Abundance, fill=factor(genus, levels=c("Alysiella","Arcobacter","Chryseobacterium","Citrobacter","Gallicola","Kocuria","Luteimonas","Mucilaginibacter","Snodgrassella","Veillonella","Other","Unknown")))) +

geom_bar(aes(),stat="identity",position="fill") +

ylab("Relative Abundance (%)") + facet_wrap(~PIT,scales = "free_x", drop = F, ncol= 1) +

theme(panel.grid.major = element_blank(), panel.grid.minor = element_blank()) +

scale_fill_manual(values=my_colors) + guides(fill=guide_legend(title="Genus")) +

theme(axis.title.x=element_blank()) + theme(axis.text.x = element_text(angle = 45, vjust = 1, hjust=1))

plot_grid(phy_ind,gen_ind)

### edgeR ####

phyloseq_to_edgeR = function(physeq, group, method="RLE", ...){

require("edgeR")

require("phyloseq")

if( !taxa_are_rows(physeq) ){ physeq <- t(physeq) }

x = as(otu_table(physeq), "matrix")

x = x + 1

if( identical(all.equal(length(group), 1), TRUE) & nsamples(physeq) > 1 ){

group = get_variable(physeq, group)

}

taxonomy = tax_table(physeq, errorIfNULL=FALSE)

if( !is.null(taxonomy) ){taxonomy = data.frame(as(taxonomy, "matrix")) }

y = DGEList(counts=x, group=group, genes=taxonomy, remove.zeros = TRUE, ...)

z = calcNormFactors(y, method=method)

if( !all(is.finite(z$samples$norm.factors)) ){

stop("Something wrong with edgeR::calcNormFactors on this data,

non-finite $norm.factors, consider changing `method` argument")

}

return(estimateTagwiseDisp(estimateCommonDisp(z)))

}

# conversion ##

phyloseq_levels <- c("GenusLevel")

phyloseq_list <- list(GenusLevel = r_physeq)

da_results_list <- list()

otu_counts <- list()

for(level in phyloseq_levels) {

da_temp <- phyloseq_to_edgeR(phyloseq_list[[level]],group="Season")

otu_counts_before <- nrow(da_temp$counts)

keep <- filterByExpr(da_temp)

da_temp <- da_temp[keep, ]

otu_counts_after <- nrow(da_temp$counts)

da_results_list[[level]] <- da_temp

cat(paste("For", level, "\n",

"OTUs before filtering:", otu_counts_before, "\n",

"OTUs after filtering:", otu_counts_after, "\n\n"))

otu_counts[[level]] <- list(before = otu_counts_before, after = otu_counts_after)

}

dge <- da_results_list[["GenusLevel"]]

# run the DA

perform_DA <- function(dge_list) {

design <- model.matrix(~ 0 + group, data=dge_list$samples)

colnames(design) <- levels(dge_list$samples$group)

dge_list <- estimateDisp(dge_list, design)

fit <- glmQLFit(dge_list, design, block=dge_list$samples$Individual)

# Define contrasts based on the unique levels in the group factor

contrast_matrix <- makeContrasts(AuSu = Autumn - Summer, SpAu = Spring - Autumn,

SuSp = Summer - Spring, levels = design)

da_results <- list(

AuSu = glmQLFTest(fit, contrast=contrast_matrix[, "AuSu"]),

SpAu = glmQLFTest(fit, contrast=contrast_matrix[, "SpAu"]),

SuSp = glmQLFTest(fit, contrast=contrast_matrix[, "SuSp"])

return(da_results)

}

da_results<-perform_DA(dge)

# Perform DA for "GenusLevel"

NCBI_genus_results <- perform_DA(dge)

sigtab <- topTags(NCBI_genus_results[["SpAu"]], n=Inf)$table

theme_set(theme_bw())

scale_fill_discrete <- function(palname = "Set1", ...) {

scale_fill_brewer(palette = palname, ...)

}

sigtabgen = subset(sigtab, !is.na(genus))

x = tapply(sigtabgen$logFC, sigtabgen$genus, function(x) max(x))

x = sort(x, TRUE)

sigtabgen$genus = factor(as.character(sigtabgen$genus), levels = names(x))

SpAu <- ggplot(sigtabgen, aes(x = genus, y = logFC, color = phylum)) + geom_point(size=4) +

theme(axis.text.x = element_text(angle = 45, hjust = 1))+

geom_hline(aes(yintercept=0),linetype='dashed') +

theme(text = element_text(size = 15)) + ggtitle("Change from Autumn to Spring")+

guides(color=guide_legend(title="Phylum")) +xlab("")+ylab("")+ guides(color="none")

# Perform DA for "GenusLevel"

NCBI_genus_results <- perform_DA(dge)

sigtab2 <- topTags(NCBI_genus_results[["AuSu"]], n=Inf)$table

theme_set(theme_bw())

scale_fill_discrete <- function(palname = "Set1", ...) {

scale_fill_brewer(palette = palname, ...)

}

sigtabgen2 = subset(sigtab2, !is.na(genus))

x = tapply(sigtabgen2$logFC, sigtabgen2$genus, function(x) max(x))

x = sort(x, TRUE)

sigtabgen2$genus = factor(as.character(sigtabgen2$genus), levels = names(x))

AuSu <- ggplot(sigtabgen2, aes(x = genus, y = logFC, color = phylum)) +

theme(axis.text.x = element_text(angle = 45, hjust = 1))+

geom_hline(aes(yintercept=0),linetype='dashed') +

theme(text = element_text(size = 15)) + ggtitle("Change from Summer to Autumn")+

guides(color=guide_legend(title="Phylum")) + xlab("")+ylab("Log-fold change")+ guides(color="none")

# Perform DA for "GenusLevel"

NCBI_genus_results <- perform_DA(dge)

sigtab <- topTags(NCBI_genus_results[["SuSp"]], n=Inf)$table

theme_set(theme_bw())

scale_fill_discrete <- function(palname = "Set1", ...) {

scale_fill_brewer(palette = palname, ...)

}

sigtabgen = subset(sigtab, !is.na(genus))

x = tapply(sigtabgen$logFC, sigtabgen$genus, function(x) max(x))

x = sort(x, TRUE)

sigtabgen$genus = factor(as.character(sigtabgen$genus), levels = names(x))

SuSp <- ggplot(sigtabgen, aes(x = genus, y = logFC, color = phylum)) + geom_point(size=4) +

theme(axis.text.x = element_text(angle = 45, hjust = 1))+

geom_hline(aes(yintercept=0),linetype='dashed') +

theme(text = element_text(size = 15)) + ggtitle("Change from Spring to Summer")+

guides(color=guide_legend(title="Phylum")) +xlab("")+ylab("")+ guides(color="none")

plot_grid(AuSu,SpAu,SuSp, nrow=1, labels=c("A","B","C"))

# core ####

ps.m3.rel <- microbiome::transform(r_physeq, "compositional")

core.taxa.standard <- core_members(ps.m3.rel, detection = 0.0001, prevalence = 80/100)

core.taxa.standard

# summer

r_summer <- subset_samples(r_physeq, Season=="Summer")

core.taxa.standard <- core_members(r_summer, detection = 0.0001, prevalence = 80/100)

core.taxa.standard

r_autumn <- subset_samples(r_physeq, Season=="Autumn")

core.taxa.standard <- core_members(r_autumn, detection = 0.0001, prevalence = 80/100)

core.taxa.standard

r_spring <- subset_samples(r_physeq, Season=="Spring")

core.taxa.standard <- core_members(r_spring, detection = 0.0001, prevalence = 80/100)

core.taxa.standard

lm <- lm(Body_condition~Season, data=sample_info_tab_r)

anova(lm)

lm <- lm(Ticks~Season, data=sample_info_tab_r)

anova(lm)

my_comparisons <- list(c("Summer","Spring"),c("Summer","Autumn"),c("Spring","Autumn"))

symnum.args = list(cutpoints = c(0, 0.0001, 0.001, 0.01, 0.05, 1), symbols = c("****", "***", "**", "*"))

ticks <- ggplot(sample_info_tab_r, aes(x=Season,y=Ticks)) + stat_n_text() + stat_compare_means(method = "wilcox.test", comparisons = my_comparisons, label = "p.signif", symnum.args = symnum.args)+

geom_boxplot(fill='#00b33c', color="black") + theme(panel.background = element_blank())+ theme(axis.line = element_line(colour = "black")) + xlab("Sites") + ylab("Number of ticks") +

theme(text=element_text(size=15))

my_comparisons <- list(c("Summer","Spring"),

c("Summer","Autumn"),

c("Spring","Autumn"))

body_condition <- ggplot(sample_info_tab_r, aes(x=Season,y=Weight)) + stat_n_text() + stat_compare_means(method = "wilcox.test", comparisons = my_comparisons, label = "p.signif", symnum.args = symnum.args)+

geom_boxplot(fill='#00b33c', color="black") + theme(panel.background = element_blank())+ theme(axis.line = element_line(colour = "black")) + xlab("Sites") + ylab("Weight (g)") +

theme(text=element_text(size=15))
